# Supplementary material for: Distinct methane-dependent biogeochemical states in Arctic seafloor gas hydrate mounds
Source: Nat Commun. 2021 Nov 2;12:6296. doi: 10.1038/s41467-021-26549-5 (PMC8563959; doi:10.1038/s41467-021-26549-5)
Supplement: Supplementary file 1 — Supplementary Information [file 41467_2021_26549_MOESM1_ESM.docx]

### **Supplemental Information for “Distinct methane-dependent biogeochemical states in Arctic seafloor gas hydrate mounds”, Klasek et al.**

#### **Modeling approach**

#### The model applied on all cores in this study is the reduced model as described previously in Hong et al, 2017^1^ and aims to quantify both AOM rates and the timing of SMT shoaling. The model considers a 60-m sediment column (the thickness of hydrate stability zone in the area) beginning with linear sulfate and methane profiles (Fig. S1, left panel). We approach the modeling with the following assumptions:

#### AOM is the only reaction responsible for sulfate consumption at these sites. The assumption is reasonable given the low concentration of ammonium measured. We measured up to ~200 μM of ammonium from the bottom of GC1045 (Fig. S2), which corresponds to 1.2 mmol of organic carbon being degraded in every liter of porewater assuming a C/N atomic ratio of 6.1^2^ (or 0.84 mmol of organic carbon per liter bulk sediment, assuming a porosity of 0.7). Though some of the cores investigated do not have ammonium data, the conclusion of insignificant organoclastic sulfate reduction is supported by 119 porewater ammonium concentration measurements over the years of investigation from the different GHMs (Fig. S2 and Table S3). Maximum ammonium concentrations from cores presented here are consistent with less than 6% of sulfate being reduced with organic matter instead of methane (Fig. S2). This small contribution of organic carbon degradation to sulfate reduction is negligible when estimating the rate of SMT shoaling.

#### The non-steady-state porewater profiles are the result of sudden increases in methane flux. GC1045 and GC1081 lie in the vicinity of sites where non-steady-state porewater sulfate profiles were attributed to increasing methane flux^1^, thus we see such an assumption is also applicable here. Briefly, Hong et al. examined the five different scenarios that have been proposed in the literature that could cause similar non-steady-state porewater profiles^1^. Irrigation either due to biological or physical disturbance in shallow sediments results in seawater-like pore water composition. The buildup of ammonium at GC1045 (an increase in >58 μM from 10 to 54 cm below seafloor) allows us to exclude this process. Irrigation enhances the mixing between oxic bottom water and pore fluid and decreases the concentration of ammonium. Advection of the aqueous phase (i.e., pore fluid) is also excluded, as this process cannot explain the profiles from all solutes, such as calcium.

#### The sudden increase in methane supply is due to methane gas dissolution: It was concluded that the high methane flux at GHM3 was due to methane gas dissolution^1,3^, likely through the fracture network system in the region. Presence of gas in the sediments was confirmed by the seismic blank zone beneath the investigated gas hydrate mound, gas hydrate recovered in the sediments, and the persistent hydroacoustic flare observed in the water column^3^. This interpretation was later supported by independent geophysical evidence^4^.

#### In the model, only the aqueous phase was simulated. Both the AOM rates and timing are essentially constrained by the diffusion of sulfate and methane. The governing equations are:

#### **Equation 1:** $\frac{dC}{dt}=-\frac{1}{\phi}\frac{dF}{dx}+R_{AOM}$

#### **Equation 2:** $F=-\phi D_{s}\frac{dC}{dx}$

#### where *φ*, *D_s_*, and $\frac{dC}{dx}$ are sediment porosity (0.7), diffusion coefficients in porous media, and concentration gradients for the two target species, *t* is time in years, *x* is depth in meters below seafloor (mbsf), *C* is the concentration of porewater species in mole/m^3^ (volume of bulk sediments), and *R_AOM_* is the AOM reaction rate in mole/m^3^/yr. Diffusion coefficients for seawater media were corrected with bottom water temperature measured during CTD casts in May 2015 (0.56^o^C) and a tortuosity of 1.5 to derive the diffusion coefficients in porous media (*D_s_* in Equation 2). We calculated 0.0072 and 0.0134 m^2^/yr for the diffusion coefficients of sulfate and methane^5^, respectively.

#### For all cases, we assigned seawater composition as the top boundary condition and used no flux boundary as the lower boundary condition for sulfate. For the simulation of steady state methane dynamics, we initiated the model with a SMT of 7.4 mbsf (left panel of Fig. S1). These initial conditions were determined by fitting the modeling results with the shallow sulfate profile from GC1045, a site with an increased methane flux. A methane concentration of 1000 mM was assigned as the lower boundary condition. We ran the model until the downcore porewater sulfate and methane concentrations did not significantly change, an interval corresponding to ~40,000 years (Fig. 2A-D). For the scenarios with a recent increase in methane supply and the transitional state, we used the same sulfate and methane profiles as initial conditions (Fig. S1). We assigned much higher concentrations of methane (5E100 mM and 100 mM for the two scenarios, respectively) at 60 mbsf to simulate the sudden increase of methane supply at these sites. These bottom methane concentrations do not represent *in situ* porewater concentrations at depth, but serve to simulate cases with different methane supplies. Under such an increased methane flux, rapid AOM stimulated by the high methane concentration consumes sulfate at rates that are much faster than the replenishing of sulfate through diffusion from the bottom water, resulting in the non-steady-state profiles observed (Fig. 2I-L). As the model does not include gas dissolution and gas hydrate dynamics, AOM rates were constrained only by the sulfate profiles. We calculated AOM numerically by discretizing depth using a centered forward finite difference scheme and time using an implicit Crank-Nicholson scheme. The depth and time discretization (*dx*=0.025 meter and *dt*=0.01 year) were determined by running the model with progressively smaller discretization until the results were numerically stable and accurate.

#### We solved the R_AOM_ term explicitly as:

#### **Equation 3:** $R_{AOM}=R_{AOM}^{max} \frac{C_{{SO}_{4}}}{C_{{SO}_{4}} + k_{{half-SO}_{4}}} \frac{C_{{CH}_{4}}}{C_{{CH}_{4}} + k_{{half-CH}_{4}}}$

#### Where $k_{{half-SO}_{4}}$ and $k_{{half-CH}_{4}}$are the half saturation constants for sulfate^6^ (0.5 mol/m^3^) and methane^7,8^ (5 mol/m^3^), respectively. $R_{AOM}^{max}$ is the theoretical maximum AOM rate obtained by fitting the sulfate profile (2 mol/m^3^/yr). The magnitude of this value affects the shape of profiles close to the SMT depth, but not the rate of SMT migration.

#### There are two freely-adjusted parameters in this model: the lower boundary condition for methane (i.e., the concentration at 60 mbsf), and the time since the methane pulse initiated. The first parameter was constrained by the SMT depth for the steady-state case and by the curvature of the sulfate profiles for the case of an increased methane flux. For the latter scenario, the methane flux must be great enough to simulate AOM that can outcompete sulfate diffusion from the seafloor. An insufficient methane flux will result in a sulfate profile that is smoother than the observed profiles^1^. With methane flux being constrained, we can estimate the duration of the methane pulse required to fit the data. Though it is not our intention to fit data from specific sites, porewater sulfate concentrations from sites investigated in the present study and previous work were plotted in Fig. 2 as a comparison. AOM rate estimates for non-steady-state sites experiencing increases in methane flux are presented in Fig. 4D and Table S2. AOM rates calculated from steady-state cores are based on model run times of 50,000 years for GCs 1068/1069/1070 (Fig. 2C-D, Fig. S4), and in the transitional state of core GC1048, 3,290 years (Fig. 2G).

####

#### *
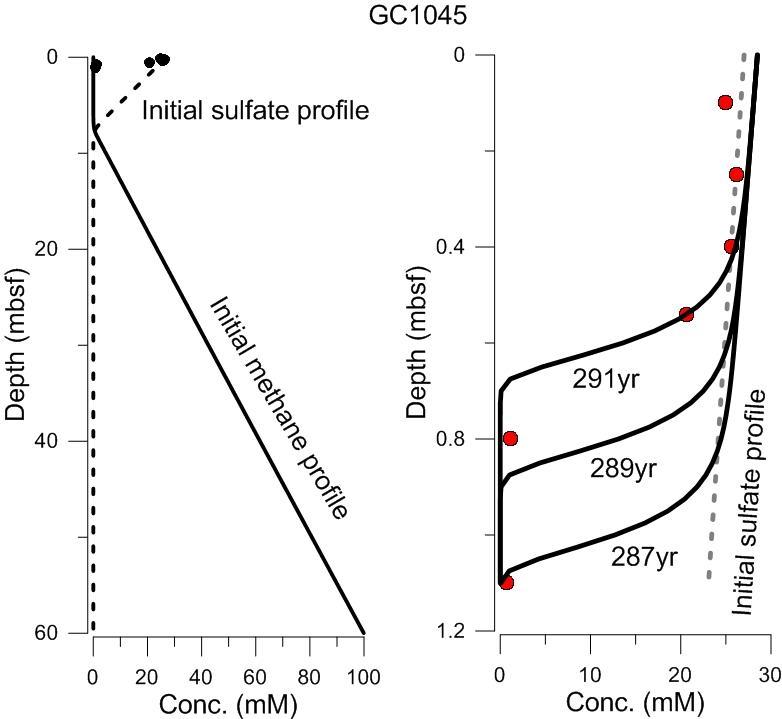
*

#### Figure S1. Initial condition and time progressing model results applied on gravity cores GC1045 and GC1081. Points show measurements of porewater sulfate. mbsf, meters below seafloor; conc, concentration.

####

####


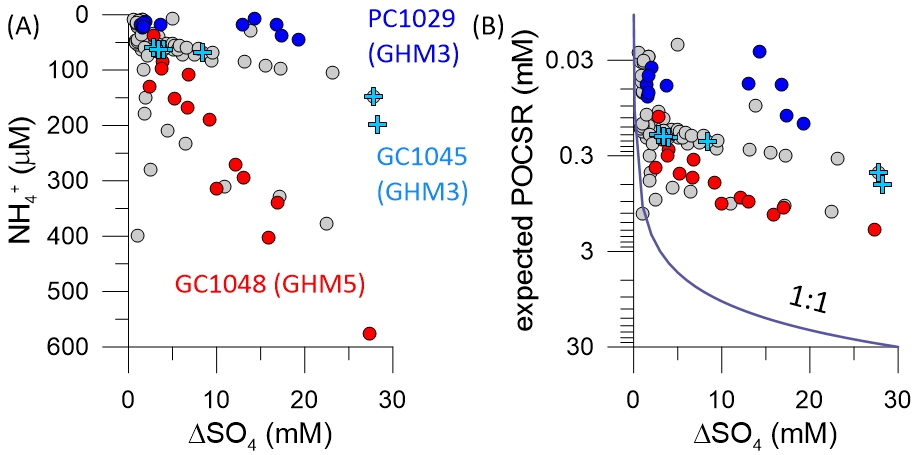


Figure S2. Porewater ammonium concentrations (A) and expected particulate organic carbon sulfate reduction (POCSR, in panel B) with respect to change in sulfate with depth. Dark blue circles are from seep push core PC1029, light blue crosses are from gravity core GC1045, red circles are from steady-state core GC1048, and gray circles are from sites investigated in previous studies (Table S3). The expected POCSR was calculated by assuming a molar C/N ratio of 6.1 in the organic matter^2^. One mole of ammonium released in the porewater thus represents 6.1 mole of organic matter and 3.05 mole of sulfate being consumed ignoring any diffusional loss of ammonium. Such an estimate is therefore conservative. A 1:1 ratio of expected POCSR/∆SO_4_ would suggest organic carbon degradation as the only sink for sulfate. The expected POCSR is no higher than 1.7 mM of sulfate, which supports anaerobic methane oxidation as the primary sink.
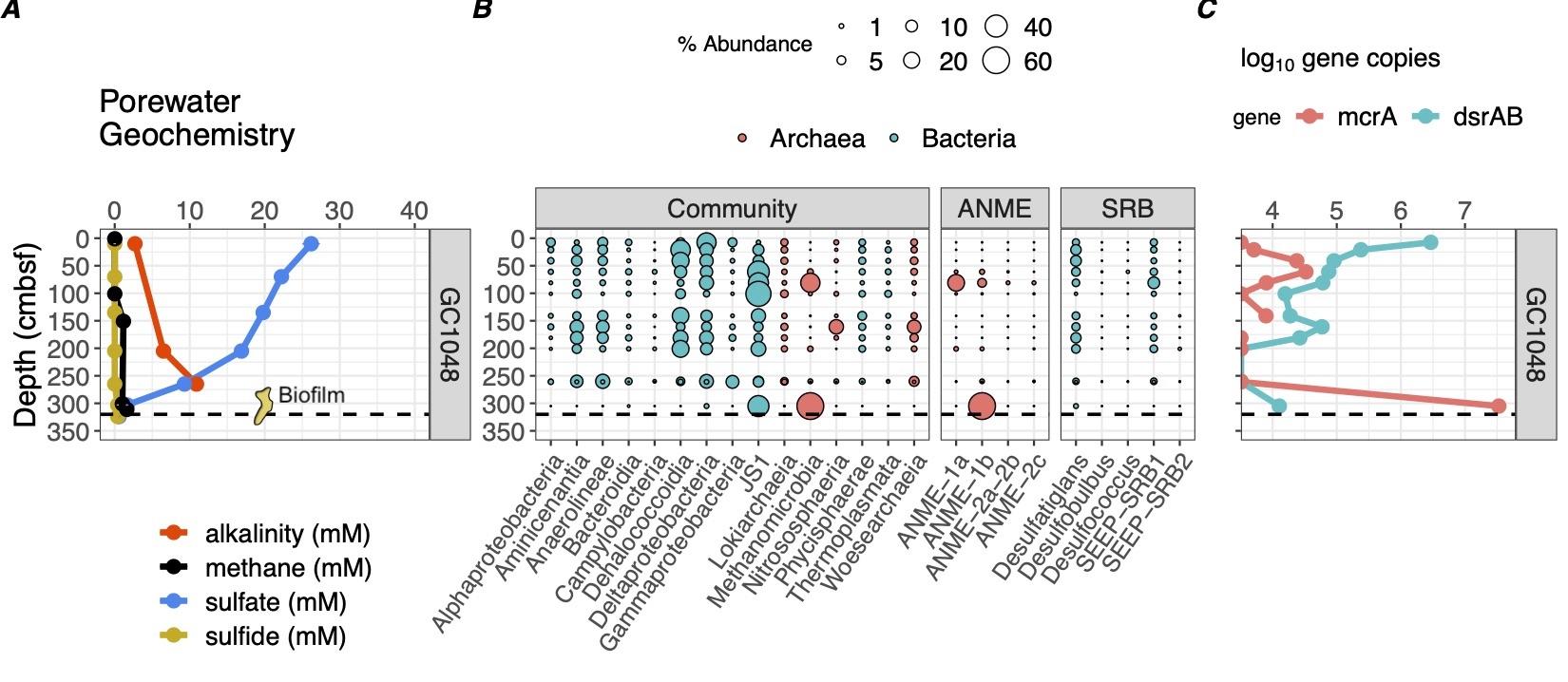
Figure S3. Geochemical, microbial community, and gene abundance data from GC1048, west of GHM3. This transitional-state core is consistent with a recent, but minor, increase in methane flux. Sulfate-methane transition depth is indicated by the dashed line. (A) shows methane concentrations and porewater sulfate, sulfide, and alkalinity, and (B) indicates percent abundances of dominant bacterial and archaeal classes, dominant anaerobic methanotrophic archaeal (ANME) families, and sulfate-reducing bacterial (SRB) genera. (C) shows copy numbers of *mcrA* and *dsrAB* genes per gram bulk sediment, with values below the detectable limit (10^3^ g^-1^) along the margin of the panel. Macroscopic translucent-to-yellow biofilms, shown as yellow symbols in panel (A), were observed at 305 cm below seafloor (symbol size not to scale with depth axis).


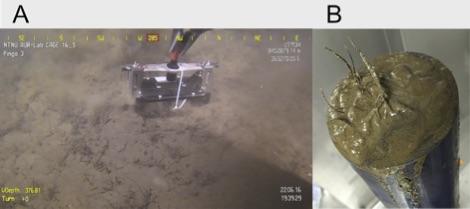


Figure S4. Siboglinid tubes identified at summit of Gas Hydrate Mound 3. Remote-operated vehicle (ROV) image of a Blade core (A) at the summit of GHM3 near where push core PC1029 was taken, showing abundant siboglinid tubes at the seafloor. A ROV-guided push core (replicate of PC1029) shows worm tubes in finer detail (B).


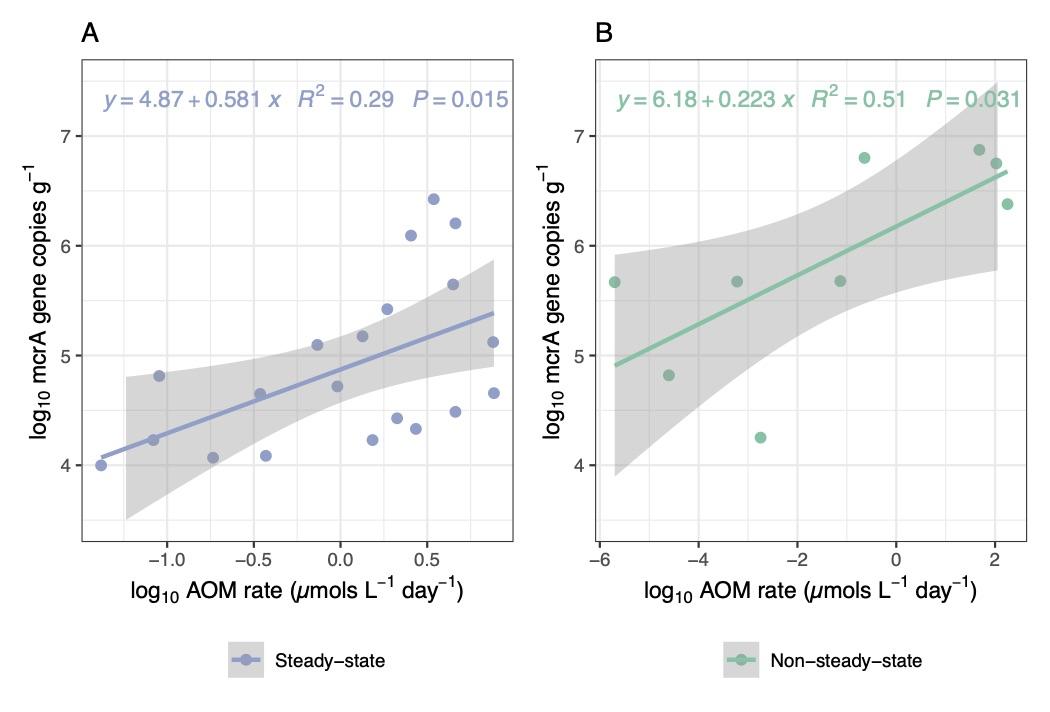


Figure S5. Linear regression of log-*mcrA* gene concentrations (copies per gram bulk sediment) to log-modeled rates of AOM, with regression *p*-value and multiple R^2^ shown. Samples that did not contain detectable *mcrA* were not included. Push core PC1029 samples were also omitted due to uncertainties in calculating AOM rates.


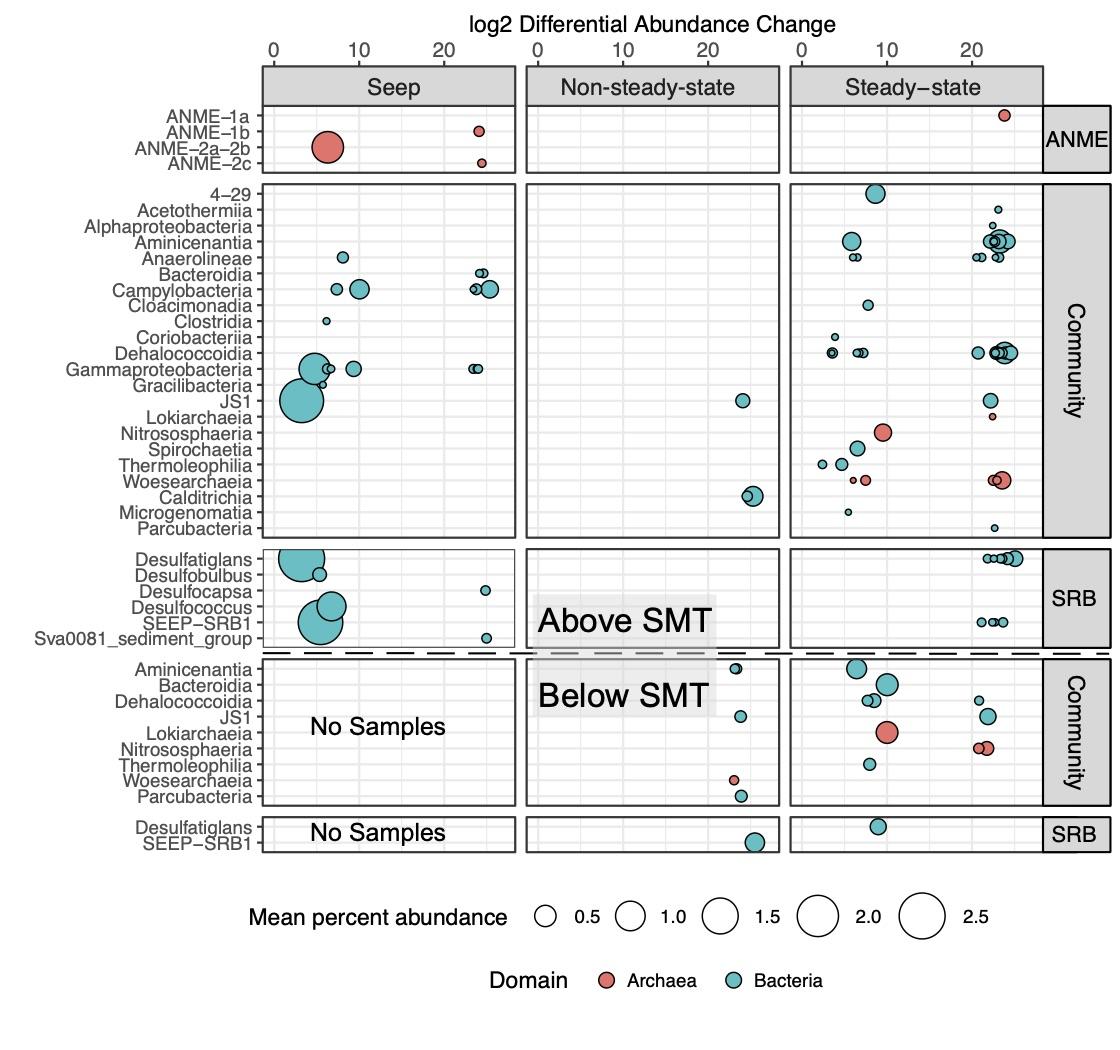


Figure S6. Differentially abundant ASVs (amplicon sequence variants) identified across three states of methane dynamics, either above or below the sulfate-methane transition (SMT, indicated by dashed line). Sizes of bubbles correspond to mean percent abundances of ASVs within the corresponding methane state. Higher differential abundance values (top axis) represent stronger statistical associations between an ASV and a particular group. In addition to position relative to the SMT, vertical panels convey class-level taxonomic annotations for all taxa except ANME (at family level) and SRB (at genus level). Bubble sizes represent mean percent abundances within each group. Differential abundance was inferred using an alpha of 0.05 and a Benjamini-Hochberg correction for multiple comparisons.


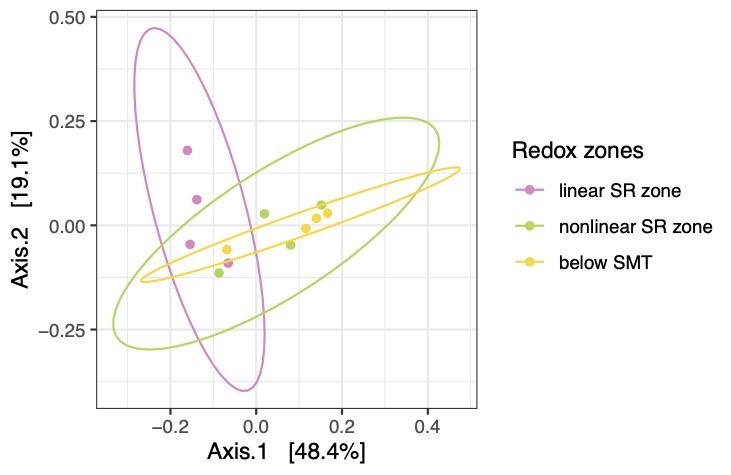


Figure S7. Principal coordinates analysis (PCoA) of communities from sites experiencing increasing methane flux suggesting linear sulfate reduction (SR) zone communities are distinct from the other two zones. PERMANOVA test between linear and nonlinear, R^2^ =0.30173, *p*=0.046; between linear and below-SMT, R^2^ =0.42094, *p*=0.023; between nonlinear and below-SMT, R^2^ =0.12485 *p*=0.435. A weighted Unifrac distance matrix with Hellinger transformation was used.

#### **Network Analysis**

Co-occurrence networks between bacterial and archaeal ASVs are often used to predict potential interactions between members of complex communities^9^, and have recently been used to infer experimentally-validated interactions among subclades of ANME and SRB^10^. To examine the extent to which ANME and SRB associate with one another in contrast to other community members in this dataset, we used FlashWeave^11^, a program that infers co-occurrences between compositional microbiome data, to construct a network of 844 ASVs that were present in at least three of the 76 total samples and whose percent abundances were over 0.01%. Weights (co-occurrence strengths between ASVs below 0.5) were omitted, and isolated nodes (ASVs with no connections to others) were removed. Fig. S7A shows the graph of all remaining ASVs as nodes colored by taxonomy (ANME, red; SRB, green; other, gray) and sized by the square root of their mean percent abundance across all samples. Fig. S7B-L are subsets of the graph that show strong connections between dominant ANME, SRB, and other ASVs. From this network, we conclude that many of the most dominant ANME and SRB show strong co-occurrences with one another that may reflect true, widely documented cell-cell interactions used for coupling AOM and sulfate reduction^12^. Similar to the findings of Metcalfe et al., 2020^10^, our network reveals high connectivity between ANME and SRB members, though in contrast, our most dominant ANME-SRB interaction (Fig. S7C) is a co-occurrence between a member of ANME-1a and SEEP-SRB1. In several instances ANME or SRB associate with other, often lower-abundance community members: associations with potential organic matter-degrading *Aminicenantales*^13^ (Fig. S7B, S7K) may suggest coupled cycles of AOM and/or SR with organic matter degradation to a lesser extent.


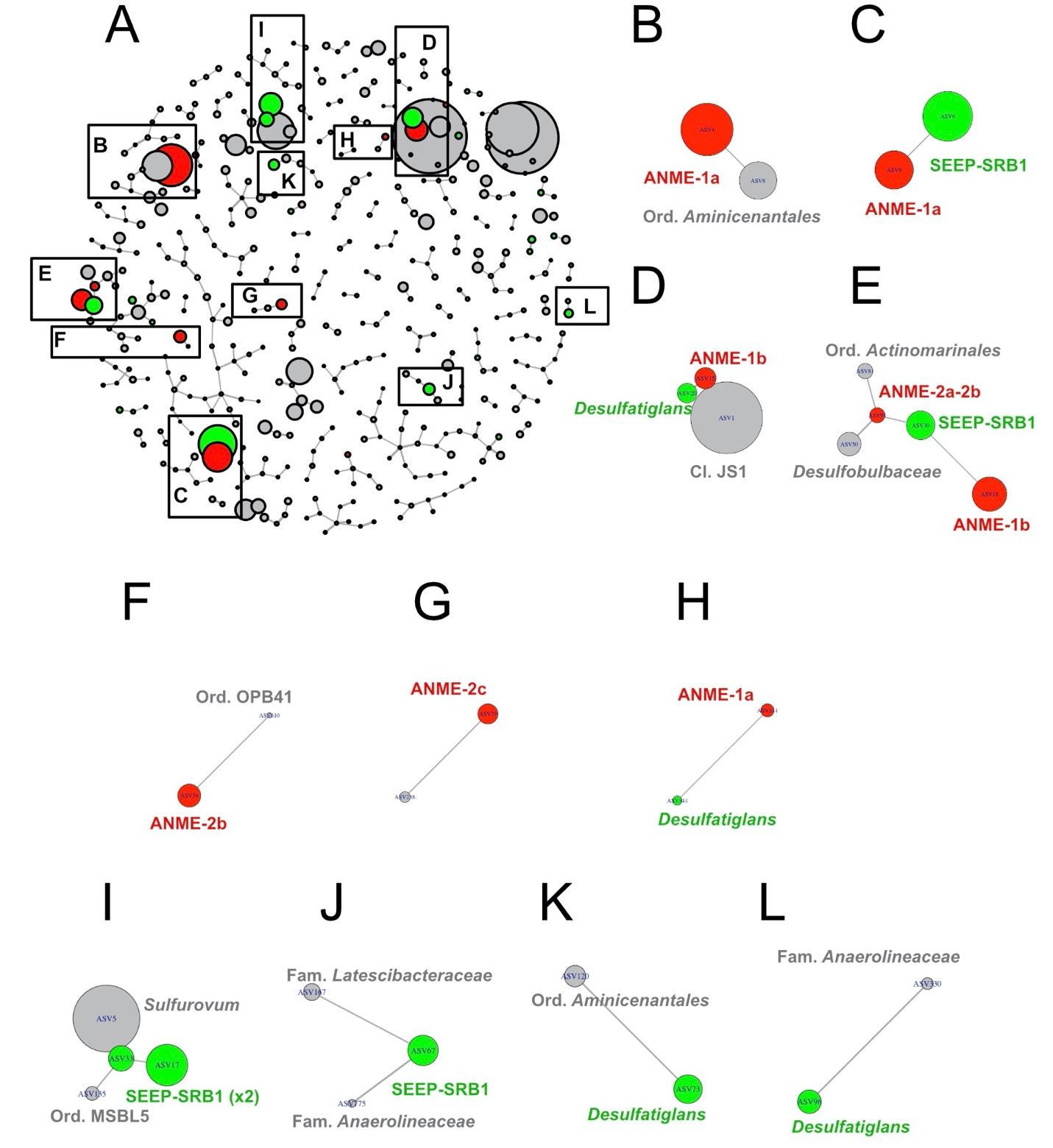


**Figure S8.** Co-occurrence network between abundant ASVs in all 76 communities analyzed in this study (including 13 from GC1048). ANME ASVs are shown in red, SRB in green, and others in gray. Nodes are sized by the square root of their mean percent abundance across all samples (A). Subsets of the graph that show strong connections between dominant ANME members and other ASVs are shown in panels B-H. The most specific taxonomies represent genera unless specified. Sizes reflect percent abundances between ASVs within a panel, but not between panels.

#### **Supplemental Tables**

| Core | Latitude | Longitude | Water depth (m) | Core recovery (cm) | SMT depth (cm) | Methane flux (mol m^-2^ yr^-1^) at time of sampling |
| --- | --- | --- | --- | --- | --- | --- |
| PC1029 | 76 06.398 | 15 58.151 | 381 | 27 | NA | * |
| GC1045 | 76 06.347 | 15 57.959 | 387 | 130 | 82 | 5.28 |
| GC1081 | 76 07.022 | 16 02.593 | 369 | 102 | 56 | 4.22 |
| GC1048 | 76 06.737 | 15 59.845 | 387 | 335 | 320 | 0.31 |
| GC1068 | 76 06.739 | 16 00.311 | 384 | 295 | 108 | 1.30 |
| GC1069 | 76 06.719 | 16 00.334 | 383 | 227 | 138 | 1.30 |
| GC1070 | 76 06.703 | 16 00.162 | 385 | 326 | 69 | 1.30 |

**Table S1.** Latitude, longitude, water depth, core recovery, sulfate-methane transition depth, and methane flux of all cores analyzed in this study. Fluxes are calculated by integrating AOM rate calculations across the depth of cores. Fluxes were derived from the steady-state scenario of modeling using results of 3,290 yrs (GC1048) and 50,000 yrs (GCs 1068/1069/1070). *Assuming the drawdown in sulfate at PC1029 is associated with AOM only, we estimate a flux on the order of 10-100 mol m^-2^ yr^-1^ at the site of active seepage.

| Core | Years before sampling | CH4 flux (mols/m2/yr) | Peak AOM depth (cm) |
| --- | --- | --- | --- |
| GC1045 | 21 | 3.674 | 280 |
| GC1045 | 20 | 3.744 | 270 |
| GC1045 | 19 | 3.808 | 257.5 |
| GC1045 | 18 | 3.874 | 247.5 |
| GC1045 | 17 | 3.944 | 237.5 |
| GC1045 | 16 | 4.021 | 227.5 |
| GC1045 | 15 | 4.101 | 217.5 |
| GC1045 | 14 | 4.184 | 207.5 |
| GC1045 | 13 | 4.268 | 197.5 |
| GC1045 | 12 | 4.351 | 187.5 |
| GC1045 | 11 | 4.433 | 177.5 |
| GC1045 | 10 | 4.514 | 167.5 |
| GC1045 | 9 | 4.593 | 157.5 |
| GC1045 | 8 | 4.671 | 147.5 |
| GC1045 | 7 | 4.749 | 137.5 |
| GC1045 | 6 | 4.826 | 127.5 |
| GC1045 | 5 | 4.902 | 117.5 |
| GC1045 | 4 | 4.978 | 107.5 |
| GC1045 | 3 | 5.054 | 97.5 |
| GC1045 | 0 | 5.279 | 67.5 |
| GC1081 | 22 | 2.93 | 275 |
| GC1081 | 17 | 3.253 | 225 |
| GC1081 | 12 | 3.589 | 175 |
| GC1081 | 7 | 3.899 | 125 |
| GC1081 | 2 | 4.207 | 75 |
| GC1081 | 1 | 4.267 | 65 |
| GC1081 | 0 | 4.325 | 55 |
| GC1081 | -1 | 4.379 | 45 |
| GC1081 | -2 | 4.43 | 35 |

**Table S2.** Increases in methane flux over the past two decades for cores GC1045 and GC1081 and corresponding depths of modeled peak AOM rates. Fluxes are integrated from AOM rate data, using cell widths of 2.5 cm. (Peak AOM depths also at 2.5 cm resolution).

| **Core ID** | **GHM NO.** | **[NH_4_]_highest_ (mM)** | **Sed. Dep. of [NH_4_]_highest_ (cm)** | **Reference** |
| --- | --- | --- | --- | --- |
| GC911 | GHM3 | 106 (n=5) | 57 | a, b, c, d |
| GC1520 | GHM3 | NA | NA | a, c |
| GC1521 | GHM3 | NA | NA | a, c, g |
| GC1522 | GHM3 (background) | NA | NA | a, c |
| GC940 | GHM3 | 406 (n=15) | 293 | a, c, d |
| GC1045 | GHM3 | 198 (n=6) | 110 | d |
| PC1029 | GHM3 | 46 (n=11) | 23 | g |
| MC904 | GHM3 | 68 (n=14) | 31 | a, b, g |
| MC932 | GHM3 | 7 (n=6) | 11 | g |
| MC938 | GHM3 | 60 (n=16) | 16 | g |
| MC1063 | GHM3 | 74 (n=9) | 35 | f, g |
| MC1064 | GHM3 | 67 (n=7) | 37 | f, g |
| GC918 | GHM4 | 38 (n=4) | 52 | c |
| GC1081 | GHM4 | NA | NA | c |
| GC920 | GHM5 | 193 (n=10) | 240 | a, c, d |
| GC1068 | GHM5 | NA | NA | d |
| GC1069 | GHM5 | NA | NA | d |
| GC1070 | GHM5 | 18 (n=1) | 236 | d, e |
| GC1048 | GHM5 (background) | 577 (n=15) | 303 | d,e |

a. Hong et al. (2017)^1^: In this work, the pore fluid geochemistry from GHMs was first documented to quantify the timing of methane pulses. It was concluded that the non-steady-state pore fluid profiles observed from GHM3 is due to sudden releases of methane gas from the reservoir ca. 160 to 1900 years ago. Such pulses of methane release cannot be explained by gas hydrate destabilization of contemporary ocean warming.

b. Serov et al. (2017)^14^: The authors proposed that the release of methane from GHMs was due to gas hydrate destabilization as a result of ice sheet dynamics.

c. Hong et al. (2018)^3^: The distinctly different pore fluid geochemistry between GHM3 and GHM5 was documented, which was attributed to the migration of different fluid phases: aqueous fluid at GHM5 and gaseous fluid at GHM3.

d. Sen et al. (2018): The different fluid migration behaviours from GHM3 and GHM5 result in the different assemblages of seafloor macro-fauna, which was attributed to the different methanotrophic activities sustained by the fluids of different phases.

e. Gründger et al. (2019)^15^: Methane-fuelled biofilms consisting mainly of ANME-1 were recovered from two cores at GHM5, which was characterized as a steady-state fluid system. This observation was interpreted to represent a constant and stable supply of methane at these locations.

f. Carrier et al. (2020)^16^: Communities of bacteria, archaea, and microbial eukaryotes in shallow sediments (< 30 cm below seafloor) from GHMs differ more by methane concentration than by distance from seepage, and community types associated with high-methane concentrations were found at GHM edges.

g. Hong et al. (2020): Through investigating the Fe and S dynamics, this papers discuss the Fe cycling in the sediments of cold seeps, which is analogue for the anoxic marine sediments from productive margins.

**Table S3.** Compilation of porewater ammonium concentration data from Storfjordrenna GHMs.

#### **Supplementary References**

1. Hong, W.-L. *et al.* Seepage from an arctic shallow marine gas hydrate reservoir is insensitive to momentary ocean warming. *Nature Communications* **8**, 15745 (2017).

2. Tamelander, T., Aubert, A. & Wexels Riser, C. Export stoichiometry and contribution of copepod faecal pellets to vertical flux of particulate organic carbon, nitrogen and phosphorus. *Mar. Ecol. Prog. Ser.* **459**, 17–28 (2012).

3. Hong, W.-L. *et al.* Variations in Gas and Water Pulses at an Arctic Seep: Fluid Sources and Methane Transport. *Geophysical Research Letters* **45**, 4153–4162 (2018).

4. Waage, M. *et al.* Geological Controls on Fluid Flow and Gas Hydrate Pingo Development on the Barents Sea Margin. *Geochemistry, Geophysics, Geosystems* **20**, 630–650 (2019).

5. Boudreau, B. P. *Diagenetic Models and Their Implementation: Modelling Transport and Reactions in Aquatic Sediments*. (Springer Berlin Heidelberg, 1997).

6. Wegener, G. & Boetius, A. An experimental study on short-term changes in the anaerobic oxidation of methane in response to varying methane and sulfate fluxes. *Biogeosciences* **6**, 867–876 (2009).

7. Nauhaus, K., Boetius, A., Krüger, M. & Widdel, F. In vitro demonstration of anaerobic oxidation of methane coupled to sulphate reduction in sediment from a marine gas hydrate area. *Environmental Microbiology* **4**, 296–305 (2002).

8. Vavilin, V. A. Estimating changes of isotopic fractionation based on chemical kinetics and microbial dynamics during anaerobic methane oxidation: apparent zero- and first-order kinetics at high and low initial methane concentrations. *Antonie van Leeuwenhoek* **103**, 375–383 (2013).

9. Layeghifard, M., Hwang, D. M. & Guttman, D. S. Disentangling Interactions in the Microbiome: A Network Perspective. *Trends in Microbiology* **25**, 217–228 (2017).

10. Metcalfe, K. S., Murali, R., Mullin, S. W., Connon, S. A. & Orphan, V. J. Experimentally-validated correlation analysis reveals new anaerobic methane oxidation partnerships with consortium-level heterogeneity in diazotrophy. *The ISME Journal* 1–20 (2020) doi:10.1038/s41396-020-00757-1.

11. Tackmann, J., Rodrigues, J. F. M. & Mering, C. von. Rapid Inference of Direct Interactions in Large-Scale Ecological Networks from Heterogeneous Microbial Sequencing Data. *cels* **9**, 286-296.e8 (2019).

12. Knittel, K. & Boetius, A. Anaerobic Oxidation of Methane: Progress with an Unknown Process. *Annual Review of Microbiology* **63**, 311–334 (2009).

13. Sharon, I. *et al.* Accurate, multi-kb reads resolve complex populations and detect rare microorganisms. *Genome Res.* gr.183012.114 (2015) doi:10.1101/gr.183012.114.

14. Serov, P. *et al.* Postglacial response of Arctic Ocean gas hydrates to climatic amelioration. *PNAS* **114**, 6215–6220 (2017).

15. Gründger, F. *et al.* Methane-fuelled biofilms predominantly composed of methanotrophic ANME-1 in Arctic gas hydrate-related sediments. *Scientific Reports* **9**, 9725 (2019).

16. Carrier, V. *et al.* The Impact of Methane on Microbial Communities at Marine Arctic Gas Hydrate Bearing Sediment. *Front. Microbiol.* **11**, (2020).
